# Inferring protein domain interactions from databases of interacting proteins

**DOI:** 10.1186/gb-2005-6-10-r89

**Published:** 2005-09-19

**Authors:** Robert Riley, Christopher Lee, Chiara Sabatti, David Eisenberg

**Affiliations:** 1 Department of Human Genetics, David Geffen School of Medicine at UCLA, University of California Los Angeles, Los Angeles, CA 90095, USA; 2 Institute for Genomics and Proteomics, University of California Los Angeles, Los Angeles, CA 90095, USA; 3 Howard Hughes Medical Institute, University of California Los Angeles, Los Angeles, CA 90095-1570, USA

## Abstract

We describe domain pair exclusion analysis (DPEA), a method for inferring domain interactions from databases of interacting proteins. DPEA features a log odds score, *E*_*ij*_, reflecting confidence that domains *i *and *j *interact. We analyzed 177,233 potential domain interactions underlying 26,032 protein interactions. In total, 3,005 high-confidence domain interactions were inferred, and were evaluated using known domain interactions in the Protein Data Bank. DPEA may prove useful in guiding experiment-based discovery of previously unrecognized domain interactions.

## Abstract 2

A new method for inferring domain interactions from databases of interacting proteins was used to deduce 3,005 high-confidence domain interactions from over 177,000 potential interactions.

## Background

Post-genomic biological discoveries have confirmed that proteins function in extended networks [1,2]. In particular, many proteins must physically bind to other proteins, either stably or transiently, to perform their functions. The functions of proteins are therefore inseparable from their interactions.

For each protein to interact with its appropriate network neighbors, highly specific recognition events must occur. Interaction specificity results from the binding of a modular domain to another domain or smaller peptide motif in the target protein [3]. For example, some cytoskeletal proteins bind to actin through their modular gelsolin repeat domains [4], and Src-homology 3 domains (SH3) bind to proline rich peptides that have a PxxP consensus sequence [5]. In the context of protein interaction, such domains and peptides act as recognition elements; we refer to these simply as 'domains'. Patterns of domain interactions are repeated within organisms and across taxa, suggesting that recognition patterns are conserved throughout biology [6]. Such patterns constitute a 'protein recognition code' [7], and it may be that many of these recognition patterns remain to be discovered.

Protein-protein interactions can be determined experimentally [8-12]. However, the specific domain interactions are usually not detected, and require further analysis to determine. It is therefore difficult to know which segment of a protein, often just a fraction of its total length, interacts directly with its biological partners. As most proteins consist of multiple domains [13], the underlying domain interactions are a largely unknown factor in the majority of known protein-protein interactions. Understanding domain recognition patterns would aid in understanding networks of proteins [14], and in applications such as predicting the effects of mutations [15] and alternative splicing events [16] that affect interaction domains, developing drugs to inhibit pathological protein interactions [17,18], and designing novel protein interactions from appropriate domain scaffolds [19].

High-throughput protein interaction studies and databases of protein interactions [8-12,20,21] present an opportunity to discover domain interaction patterns through statistical analysis of domain co-occurrence in interacting proteins. The idea is to find pairs of domains that co-occur significantly more often in interacting protein pairs than in non-interacting pairs.

However, such bioinformatic discovery of domain interaction patterns is complicated by the lack of data on which protein pairs interact and which do not. Previously described [22-25] work in correlating domain or motif pairs with the interaction of proteins have analyzed data from genome-scale interaction assays of a single organism, usually *Saccharomyces cerevisiae*. Such exhaustive assays measure which protein pairs interact, and which do not; rigorous statistical methods to analyze these datasets have been described [24,25]. These methods can be extended beyond the scope of single proteomes to infer domain interactions from the incompletely mapped interactomes of multiple organisms such as those described in the Database of Interacting Proteins (DIP) [20,26]. Databases such as DIP are appealing because they record information from many species (DIP describes 46,000 protein interactions from over 100 organisms). Extensions to existing computational methods are therefore needed to incorporate the available wealth of evidence for domain interactions, without being unduly hindered by the limited data from proteome-wide interaction screens.

Another problem in inferring domain interactions from protein interaction data is that the most probable domain interactions tend to be the most promiscuous, or least specific, interactions. Previous methods correlated pairs of domains by their frequency of co-occurrence in interacting protein pairs [23,27,28], or by their probability of interaction [24]. However, such methods may preferentially identify promiscuous domain interactions because they screen for those that occur with the highest frequency. For an arbitrary domain *i*, many paralogs are typically found within the proteome of an organism; each may interact with a specific paralog of domain *j*. Because of the need for fidelity in cellular circuitry, members of domain families *i *and *j *do not interact promiscuously. In such cases the propensity of interaction between domain families is expected to be low, as a random member of domain family *i *will be unlikely to interact with a random member of domain family *j*. Such a domain interaction, while of obvious biological importance, will be assigned a low score by methods that detect domain interactions by their probability of interaction. Methods are therefore needed to detect these low-propensity, high-specificity domain interactions.

**Figure 1:**
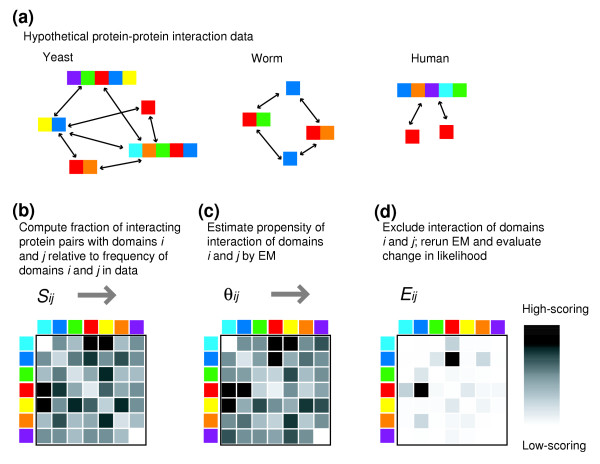
Overview of DPEA method. **(a) **In this hypothetical protein interaction dataset, domains are represented as colored squares; proteins are represented as collections of one or more domains joined together; and protein interactions are shown as black double arrows. The protein interactions are known, the domain content of each protein is known, and domain interactions are unknown. Any pair of domains that co-occur in a pair of interacting proteins is considered a potentially interacting domain pair. **(b) **The frequency of proteins with domain *i *interacting with proteins with domain *j*, *S*_*ij *_is computed. **(c) **Using *S*_*ij *_as an initial guess, the propensity, *θ*_*ij*_, of each kind of potential domain interaction is estimated by EM. **(d) **The evidence, *E*_*ij*_, for each inferred domain interaction is then assessed by calculating the change in likelihood when a given type of domain interaction is excluded.

We describe a statistical approach called domain pair exclusion analysis (DPEA) (Figure 1) to infer domain interactions from the incomplete interactomes of multiple organisms. DPEA extends earlier related methods [23,24,27,28], and adds a likelihood ratio test to assess the contribution of each potential domain interaction to the likelihood of a set of observed protein interactions. DPEA consists of three steps: (i) compile protein interaction data and compute *S*_*ij *_the frequency of interaction of each domain pair *i *and *j*, relative to the abundance of domains *i *and *j *in the data [23,27,28], (ii) using *S*_*ij *_as an initial guess, apply the expectation maximization (EM) algorithm [29] to obtain a maximum likelihood estimate of *θ*_*ij*_, the probability of interaction of each potentially interacting domain pair *i *and *j *evaluated in the context of any other domains occurring in the same proteins as domains *i *and *j *[24], and (iii) exclude all possible interactions of domains *i *and *j *from the mixture of competing hypotheses, rerun EM, evaluate the change in likelihood, and express this as a log odds score, *E*_*ij*_, reflecting confidence that domains *i *and *j *interact. A high *E*_*ij *_indicates that there is extensive evidence in protein interaction data supporting the hypothesis that domains *i *and *j *interact; a low *E*_*ij *_suggests that competing hypotheses (other potential domain interactions) are roughly as good at explaining the observed protein interactions. Application of DPEA to a small hypothetical protein interaction network is illustrated in Figure 1.

We show that domain pairs inferred to interact with high *E *are significantly enriched among domain pairs known to interact in the Protein Data Bank (PDB) [30,31], demonstrating DPEA's ability to identify physically interacting domain pairs. DPEA can also infer highly specific domain interactions by screening for domain pairs with a low *θ* and high *E*. Lastly, we explored DPEA's ability to discover previously unrecognized domain interactions by screening for interactions with high *E *involving domains with unknown function. Two examples supported by experimental evidence from the literature, involving G-protein complexes and Ran signaling complexes, are presented. These results suggest that DPEA can be used to mine protein interaction databases for evidence of conserved, highly specific domain interactions.

## Results

In total, 177,233 potential domain interactions were defined from the July 2004 release of DIP. We used the description of domain families in the Pfam database of Hidden Markov Model (HMM) profiles [32]. All DIP proteins were annotated with Pfam-A and Pfam-B domains (see Materials and methods). Proteins that could not be mapped to at least one Pfam domain, and any interactions involving such proteins, were discarded. This resulted in a dataset of 26,032 protein-protein interactions among 11,403 proteins from 68 different organisms. Our data has 12,455 distinct kinds of Pfam domains, 79% of which are of unknown function (either Pfam-B, DUF or UPF domains [32]), yielding 177,233 possible kinds of domain-domain interactions from co-occurrence of domain pairs in pairs of interacting proteins. The numbers of proteins and interactions used per organism are given in Additional data file 1; proteins and their interactions are given in Additional data files 2 and 3, respectively; protein-to-domain mappings are given in Additional data file 4.

In analyzing data from 68 organisms we assumed that pairs of domain families have the same interaction propensity across all of the organisms in which they are found. This assumption allowed us to pool multi-species interaction data for simultaneous analysis.

The interactomes of only three organisms (yeast, fly and worm) had been probed by genomewide experiments documented in the July 2004 release of DIP [8-11]. Thus the interactomes of most of the organisms documented in DIP are highly incomplete. Also, DIP does not record negative interactions, which play an important role in statistical methods for inferring domain interaction propensities [24,25]. To overcome this limitation, we made the simplifying assumption that any given pair of proteins among those in our study does not interact unless such an interaction is documented in DIP. Because all existing protein interactions are obviously not yet documented in DIP, this assumption is incorrect in some cases. However, these cases can safely be considered a small minority: the probability of two random proteins in a proteome interacting is quite small. For example, in an organism with 6,000 proteins, each with an average of four interacting partners, the probability of interaction for a random pair of proteins would be around 10^-3^. Thus in roughly 1 out of 1,000 cases, we incorrectly assume that an unreported interaction is a true negative. In summary, we assumed that: (i) observed protein interactions are true positives, (ii) unobserved protein interactions are true negatives, and (iii) any pair of proteins not both belonging to the same organism cannot interact.

The DPEA algorithm was applied to evaluate the evidence for each of the 177,233 potential domain interactions. All species for which we had domain and interaction information in DIP were analyzed simultaneously. Previous methods [23,27,28] suggested measures of domain-domain correlation based on domain pairs' frequency of co-occurrence in interacting protein pairs. We calculated a similar measure here, and called it *S*_*ij*_, an estimate of the probability of interaction between domains *i *and *j*. From *S*_*ij *_and the domain content of all interacting proteins, we estimated the likelihood of the set of observed protein interactions (see Materials and methods). We used the numerical method of EM [29], in a manner similar to [24] to maximize this likelihood and thus refine our estimate of the probability that domain *i *interacts with domain *j*, which we denote as *θ*_*ij*_, the propensity of interaction of domain *i *with domain *j*. We then performed a likelihood ratio test for each kind of domain pair by rerunning EM with all instances of that potentially interacting pair given a *θ*_*ij *_of zero, thus excluding it from the mixture of competing hypotheses. We call this score *E*_*ij*_, a measure of the evidence that domain *i *interacts with domain *j*. In total, 3,005 domain pairs had *E *scores >3.0 (Additional data file 5), corresponding to an approximate 20-fold drop in probability upon exclusion of all possible instances of the domain interaction from the set of observed protein interactions. Likelihoods in the *E *score were calculated only from positive interactions: negative or unknown interactions were not considered.

The 50 domain pairs with the highest *E *scores are shown in Table 1. Table 1 also shows statistics on the average modularity (*m*) and number of occurrences (*n*) of each kind of domain in DIP. In particular, modular domains are of considerable interest for their role in protein interactions [3]. Assessment of domain modularity therefore allows distinction of the interactions of modular domains from the interactions of domains that only occur as single-domain proteins (which DPEA assigns a high *E *score due to the lack of competing domain interactions). Of the 3,005 inferred domain interactions with *E *score >3.0, 1,510 or about 50% involve domains with *m *≥ 2.0. Table 1 suggests that the inferred domain interactions with the highest *E *score typically occur between domain families that are present in multiple occurrences in DIP. In fact, a high *E*_*ij *_correlates with an increase in the minimum number of occurrences of domains *i *or *j *(correlation coefficient = 0.019, *P *value << 0.001).

**Figure 2:**
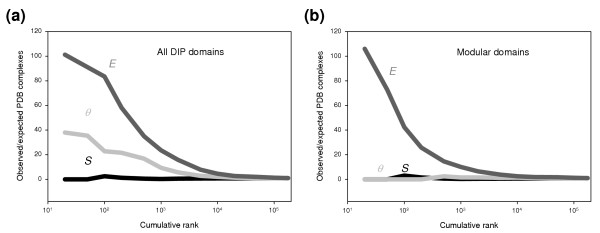
Enrichment of PDB complexes in highest-ranking domain pairs predicted to interact. Ratio of observed/expected PDB complexes in each sample of domain pairs is plotted against cumulative rank. For example, the top 100 domain pairs ranked by *E *have 71-fold more PDB complexes than would be expected in 100 randomly chosen potentially interacting domain pairs in DIP. Potentially interacting domain pairs were ranked by each of three measures: *S*, *θ* and *E*. **(a) **Ranking all domain pairs by their frequency of co-occurrence in interacting protein pairs, *S*, yielded no significant enrichment of PDB complexes at any rank cutoff. A significant enrichment of PDB complexes was seen when domain pairs were ranked by *θ*, and even more so ranked by *E*, as shown by the successive increase in observed/expected PDB complexes at each cumulative rank. The ratio using all three measures approaches 1.0 as the number of ranked complexes approaches total number of predictions in the dataset. Our results suggest that the *E *score output by DPEA performs better than *S *or *θ* at identifying physically interacting domain pairs. **(b) **Ranking interactions of modular domains by *E *reveals enrichment of PDB complexes. No enrichment is found when interactions are ranked by *θ* or *S*.

DPEA preferentially assigns high *E *scores to physically interacting domains. This was determined by training DPEA on the multispecies DIP dataset with all 230 interactions solely derived from X-ray diffraction experiments removed, and validating with the set of Pfam-A domains known to directly interact in experimentally determined structures of protein complexes in the PDB [30] as defined in the iPfam database [33]. There was no significant enrichment for PDB complexes among domain pairs ranked by their *S *score at any percentile rank. EM optimization enriches for known structural complexes in the top pairs ranked by *θ* (a 1.4-fold increase over random in the top 10%, *P *value < 0.001), confirming that the *θ* is a more accurate measure of domain interaction propensities than *S*. Ranking by *E *increased the enrichment of PDB-confirmed complexes further (2.9-fold enrichment in the top 10%, *P*-value << 0.001) (Figure 2a). PDB complexes were 12 times more abundant among the 2,920 domain pairs inferred to interact with *E *scores > 3.0 (*P *value << 0.001) compared with random. We also analyzed a yeast-only subset of this data, and found a significant enrichment of PDB complexes when ranked by *E *(2.8-fold enrichment in the top 10%, *P *value << 0.001), but no enrichment when domain pairs were ranked by *S *or *θ*. We conclude that the *E *score output by DPEA is a better indicator of domain interaction, in both single and multispecies protein interaction datasets, than either *θ* or *S*.

Many of the domains in Table 1 have an average modularity (*m*) of around 1.0, suggesting that these domains tend to occur as the only domain in a protein. To ensure that DPEA doesn't simply assign high *E *scores to the interactions of non-modular domains, we performed the same PDB validation test on a set of inferred domain interactions from which inferred domain interactions not involving a modular domain were excluded. We defined a modularity threshold of *m*_*i *_≥ 2, implying that domain *i *usually occurs in combination with other domains in the same protein. Validating the filtered set of domain interactions using the iPfam database of domain-domain interactions in the PDB confirmed that DPEA assigns high *E *scores and low *S *and *θ* scores to the interactions of modular domains in DIP (Figure 2b). This trend is even more pronounced than in Figure 2a; this demonstrates that *E *is the parameter of choice for identifying modular domain interactions, and that many high-*θ* complexes are derived from the interactions of single-domain proteins.

As a control, we defined sets of known interacting and putative non-interacting domain pairs to test whether DPEA also assigns high *E *scores to domain pairs that co-occur in interacting PDB complexes, but which do not directly interact. iPfam tables were used to define 295 directly interacting domain pairs and 265 non-interacting domain pairs (see Materials and methods). While it is impossible to say that our defined set of non-interacting domain pairs never interact in nature, it is likely that this set consists of domain pairs not functionally linked via their interaction. We therefore consider these domain pairs a putative set of negatives.

Direct interaction correlates with a high *E *score (correlation coefficient = 0.023, *P *value << 0.001). No significant correlation was observed between non-interaction and high *E *score (correlation coefficient = 0.0014, *P *value = 0.56). We found a significant enrichment of interacting domain pairs among those with *E *> 3.0 (3.6-fold relative to random, *P *value << 0.001). Non-interacting domain pairs were 1.6-fold enriched among domain pairs with *E *> 3.0 relative to randomly ordered domain pairs. The enrichment of the non-interacting set was not significant, however (*P *value = 0.15). DPEA therefore assigns high *E *scores to directly interacting domain pairs at roughly 2.3 (3.6/1.6) times the rate for non-interacting domain pairs. From these rates we estimate a positive predictive value of 3.6/(3.6 + 1.6) or about 70%. We therefore conclude that around 70% or approximately 2,100 of our 3,005 high-confidence predictions are probable true positives and that around 30% or approximately 900 may be false positives. Of the 1,510 predictions involving modular domains, we estimate around 1,060 true positives and around 450 false positives.

We found that inferred domain interactions with high *E *scores are likely to be derived from multiple observed protein interactions. Of the 177,233 potentially interacting domain pairs in DIP, 88% derive evidence from only a single protein interaction. The other 12% are inferred from multiple protein interactions. A high *E *score correlated with a domain interaction being derived from multiple (at least two) protein interactions (correlation coefficient = 0.057, *P *value << 0.001). In fact, 100% of domain interactions with *E *> 7.0 were derived from multiple observations (*P *value << 0.001). Thus, *E *scores tend to increase with the amount of evidence supporting a given domain interaction.

## Discussion

**Figure 3:**
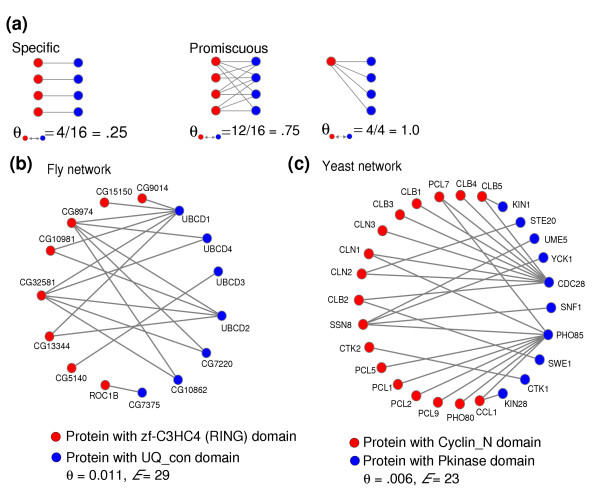
DPEA detects high-specificity domain interactions. **(a) **Interactions between domain families, such as the hypothetical red and blue domain families, whose members interact specifically are expected to have a low propensity, *θ*, because the number of interactions occurring between the domain families is a small fraction of the possible interactions (four out of 16 for two domain families of four members each). Conversely, domain interactions with a high *θ* will typically be between families whose members interact promiscuously. Because high-specificity domain interactions are of obvious interest to biologists, screening for domain interactions by their *θ* values fails to detect many important domain interactions. **(b) **Specific interactions of RING ubiquitin ligase domains [Pfam:PF00097, zf-C3HC4] with ubiquitin-conjugating enzymes [Pfam:PF00179, UQ_con] [32] in a fly protein network. The inferred domain interaction has a low *θ* (*θ* = 0.011, bottom 10%) and high *E *(*E *= 29, Table 1). This reflects the abundant evidence that the domains zf-C3HC4 and UQ_con interact, despite the low probability of interaction between any pair of these domains. **(c) **Specific interactions of Cyclin N-terminal domains [Pfam:PF00134, Cyclin_N] and protein kinase domains [Pfam:PF00069, Pkinase]. This interaction has a *θ* of 0.006, which is in the bottom 6% of *θ* for all domain pairs, suggesting the low propensity of interaction among members of these two domain families. However, the *E *score of 23 (the 13th highest score in the database) reveals the high degree of evidence for the Cyclin_N ↔ Pkinase interaction. These results show that DPEA identifies high-specificity domain interactions not detected by screening for the most probable domain interactions.

The evidence measure, *E*, detects specific domain interactions that are not detected by screening for the most probable domain interactions [23,24,27,28]. We consider *θ*_*ij *_roughly equivalent to the probability of interaction of domains *i *and *j*. If many members of domain family *i *interact non-specifically with many members of domain family *j*, we would expect a high *θ*_*ij*_, and these interactions should be easily detected by screening for those with the highest *θ*. On the other hand, if members of family *i *interact only with specific members of family *j*, we would expect a low *θ*_*ij *_(Figure 3a). Methods that screen for the most probable domain interactions therefore fail to detect highly specific domain interactions.

We find that highly specific domain interactions can be detected by screening for low *θ* and high *E*. Of the 3,005 high-confidence domain interactions (those with *E *> 3.0) we predict the 10% with highest *θ* to be promiscuous interactions; these have *θ* > 0.67. We predict the 10% with lowest *θ* to be specific; these have *θ* < 0.033. Table 1 shows several examples of inferred domain interactions with high *E *and low *θ*. For example, the known interaction of the modular RING ubiquitin ligase domains [Pfam:PF00097, zf-C3HC4] with ubiquitin-conjugating enzymes [Pfam:PF00179, UQ_con] [34] has a *θ* well below median (*θ* = 0.011, bottom 2% of high-confidence interactions), but has the eighth-highest *E *score of all potentially interacting domains in DIP (*E *= 29, Table 1). As another example, Cyclin N-terminal domains [Pfam:PF00134, Cyclin_N] are known from structural studies [PDB:1QMZ] [35] to interact with protein kinase domains [Pfam:PF00069, Pkinase]. This interaction has a *θ* of 0.006 (in the bottom 1% of high-confidence interactions) and an *E *score of 23 (13th highest, Table 1). For both zf-C3HC4 ↔ UQ_con and Cyclin_N ↔ Pkinase interactions, members of these families are expected to interact specifically to maintain fidelity of intra- and extracellular signaling. Thus our results are consistent with biological intuition. These biologically important domain interactions would not have been detected by screening for high *θ*, as the *θ* for these interactions are well below the average values for all potentially interacting domains. We therefore conclude that DPEA detects highly specific domain interactions, by high *E *and low *θ*, that are lost when domain-domain correlations are expressed as probabilities.

A potential problem in using low *θ* and high *E *to identify specific domain interactions may arise from high false negative rates of interaction datasets. Von Mering *et al*. estimated that for *Saccharomyces cerevisiae* the number of known interactions may be only a third of the number of true interactions [36]. We define specificity using non-interactions; however some of these may be false negatives. To assess how false negatives might affect our inference of specific domain interactions, we ran DPEA on a yeast-only DIP dataset (Additional data file 6), and an 'augmented' yeast dataset with randomly assigned additional interactions between proteins with Cyclin_N domains and proteins with Pkinase domains (Additional data file 7). Using the estimate of von Mering *et al*. as a guideline, we augmented the number of interactions between these two classes of proteins from 26 up to 78, thus tripling the number of potential Cyclin_N ↔ Pkinase interactions. We then ran DPEA on the unmodified yeast set and the augmented yeast set to estimate *θ* and *E *for the Cyclin_N ↔ Pkinase interaction. This resulted in an increase from *θ* = 0.015 (bottom 9%) in the augmented set up from *θ* = 0.008 (bottom 4%) in the unmodified yeast set. This suggests that, while adding missing interactions may increase *θ* for some domain interactions, for the Cyclin_N ↔ Pkinase interaction, *θ* remains low. *E *increased from 18 in the yeast reference set to 34 in the augmented set, implying that our confidence in the Cyclin_N ↔ Pkinase domain interaction would be increased by additional evidence in the form of as-yet unknown protein interactions. Additionally, 22 of 26 (85%) of the DIP interactions between proteins with these two kinds of domains have been reported in small-scale experiments, suggesting that yeast cyclins and the kinases they interact with have been relatively well-studied by experiment, and that the fraction of unknown interactions among this group of proteins may be somewhat less than for less-studied proteins. We conclude that DPEA can identify specific domain interactions even in the case of incompletely probed interactomes.

To assess the ability of DPEA to identify novel domain interactions, we analyzed inferred domain interactions that involve at least one Pfam domain of uncharacterized function. The Pfam 14.0 database contains 7,459 curated, manually annotated 'Pfam-A' domains, and 107,460 automatically generated, unannotated 'Pfam-B' domains. Because Pfam-B domains are automatically generated, and are not manually annotated, they are considered of lower information content than Pfam-A domains. In addition to Pfam-B domains, 1,503 domains in the Pfam 14.0 release begin with the prefix 'DUF' or 'UPF', signifying domains of uncharacterized function. Thus, about 95% of the domains in the combined Pfam-A and -B databases are of uncharacterized function. Many of these domains probably participate in protein-protein interactions. Of the potentially interacting domain pairs we analyzed in DIP, 1,294 involve at least one Pfam-B, DUF or UPF domain and have *E *scores greater than the significance threshold of 3.0. Because PDB complexes, when available, provide an unambiguous validation of domain interactions, we again examined the PDB for co-occurrences of inferred interacting domain pairs involving an uncharacterized domain. Where co-occurrence was found, the structures were individually inspected to identify the physically interacting protein regions. Where domains were found to interact physically, the published biochemical literature was searched further to verify the biological significance of the domain interaction.

**Figure 4:**
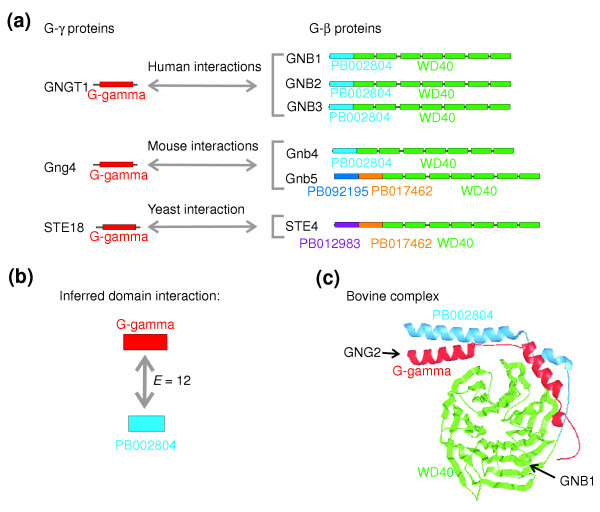
Inferred domain interactions of G-protein subunits. **(a) **Domain structures of interacting G-γ and G-β proteins in human, mouse and yeast. Protein names are in black to the left of each protein's domain structure schematic. Domains of proteins are colored boxes connected by a gray line. Pfam-A domain names and Pfam-B accession numbers are the same color as the domains they label. Domain structures are schematic and are not to scale. **(b) **Of the possible domain interactions, only that of G-gamma [Pfam:PF00631] and a Pfam-B domain [Pfam:PB002804] is inferred with high confidence (*E *= 12). **(c) **A published structure of complexed G-protein γ and β subunits [PDB:1GP2] [37] confirms our prediction that the G-gamma and PB002804 domains can interact.

DPEA identified domain interactions important for the assembly of G-protein βγ complexes. DIP describes the interactions of G-γ and G-β subunits in human, mouse and yeast (Figure 4a). G-γ proteins belong to the G-gamma domain family [Pfam:PF00631]. The G-β proteins in DIP consist mainly of WD40 domains [Pfam:PF00400] with varying Pfam-B domains as their N-terminal segments [Pfam:PB002804, PB092195, PB017462]. The possible Pfam domain interactions in these βγ complexes are shown in Table 2. Of these, only the interaction of G-gamma and PB002804 (*E *= 12) is predicted with high confidence to occur in the analyzed βγ complexes (Figure 4b). This is the highest propensity domain interaction (*θ* = 0.83) of the 177,233 potential domain interactions defined in DIP. To confirm that G-gamma and PB002804 do interact, we looked for co-occurrence of these domains in PDB complexes, and found that these domains interact in the bovine G-αβγ complex [PDB:1GP2] [37] (Figure 4c). Additionally, the G-gamma ↔ PB002804 domain interaction is supported by experimental studies demonstrating that the N-terminal peptides of G-β proteins are essential for their interactions with G-γ proteins [38,39], and that mutations or deletions in these regions abolish the formation of βγ complexes. The structure of the bovine complex shows that the WD40 domains also contact the G-gamma domains; our method does not detect this domain interaction, probably because of the large number of proteins that contain WD40 domains but do not interact with G-γ proteins. The high *θ* of this domain interaction suggests that G-β and G-γ subunits that have these domains may interact promiscuously; indeed, cross-reactivity of G-β and G-γ proteins has been demonstrated [40]. We conclude that DPEA identified a domain interaction, involving an uncharacterized domain, important for the association of G-β and G-γ proteins.

**Figure 5:**
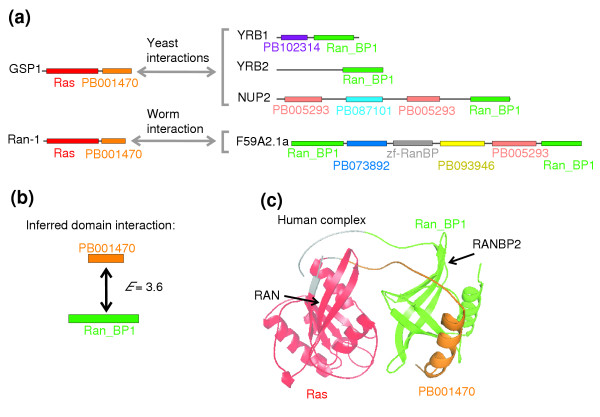
Domain interactions of Ras family members with nuclear pore proteins. **(a) **Yeast and worm Ran signal-transducing proteins interact with proteins that have Ran-binding domains [Pfam:PF00638, Ran_BP1], often found as components of nuclear pore complexes. Domain structures of the relevant interacting proteins are shown. Domains of proteins are colored boxes connected by a gray line. Protein names are in black to the left of each protein's domain structure schematic. Pfam-A domain names and Pfam-B accession numbers are the same color as the domains they label. Domain structures are schematic and are not to scale. **(b) **We find evidence for the interaction of a Pfam-B domain [Pfam:PB001470] the Ran_BP1 domain (*E *= 3.7). **(c) **Structural evidence [PDB:1RRP] [43] confirms that the domains PB001470 and Ran_BP1 interact, consistent with our prediction.

DPEA is also able to identify domain interactions important for the association of Ran signaling proteins with Ran-binding proteins. Ran proteins are members of the Ras family of GTPases [Pfam:PF00071] [41], are conserved in eukaryotes, and are important for protein transport in and out of nuclei [42]. DIP documents the interactions of yeast and worm Ran homologs with several proteins that contain a Ran-binding domain [Pfam:PF00638, Ran_BP1] (Figure 5a). The potential domain interactions underlying these protein interactions are listed in Table 3. Because of the heterogeneous domain composition of proteins that contain Ran_BP1 domains, many domain interactions are possible in this subnetwork of proteins. From among these possibilities, DPEA only detects significant evidence for the interaction of a Pfam-B domain [Pfam:PB001470] with the Ran_BP1 domain (*E *= 3.6, Figure 5b). PB001470 is unique to the Ran subfamily of Ras homologs, and is found C-terminal to the conserved Ras GTPase domain. The Ran_BP1 domain is typically found in multidomain nuclear pore complex components. The structure of human Ran complexed with the Ran-binding domain of the nuclear pore protein RanBP2 [PDB:1RRP] [43] provides unambiguous structural evidence that PB001470 interacts directly with Ran_BP1 (Figure 5c). Additional evidence for this domain interaction comes from biochemical studies showing that deletion of Ran C-terminal residues abolishes the interaction of Ran with RanBP1, a Ran effector that is homologous to the Ran-binding domain [Pfam:Ran_BP1] of RanBP2 [44]. The evidence used to infer the PB001470 ↔ Ran_BP1 interaction comes from yeast and worm protein interactions, whereas the structural and biochemical confirmation of the domain interaction is from studies of human proteins not in our DIP training set at the time of this study, suggesting that this domain interaction is phylogenetically conserved. We conclude that DPEA infers domain interactions, involving a functionally uncharacterized domain, between Ran homologs and Ran-binding proteins.

## Conclusion

A future implementation of DPEA could aim to characterize rigorously the false positive and negative rates inherent in protein interaction data. In particular, the data in DIP could be used to model a coverage probability, that is, the probability that an existing protein interaction is reported, across organisms. A false positive rate that differs across experimental methods could also be modeled. Modeling error rates in protein interaction data is of clear importance for the purpose of inferring domain interactions [24,25]. Given the computational burden posed by modeling experimental error, we chose to carry out a simpler investigation to assess the information content in DIP, and its potential for inferring domain interactions.

However, the current implementation of DPEA probably has some robustness to experimental error. We demonstrated that our estimates of *θ* and *E *would be minimally perturbed, even if the known number of protein interactions potentially occurring through the interaction of the Cyclin_N and Pkinase domains is one third the true number. DPEA may also be resilient to false positive protein interactions. False positive protein interaction data probably result from experimental artifacts, not from biologically relevant domain-domain or domain-peptide interactions. False positives will therefore tend to occur among random pairs of proteins whose constituent domains do not normally interact. High *E *scores for inferred domain interactions depend on evidence from multiple observed protein interactions. Assuming that false positives occur randomly, it is unlikely that several instances of a protein with domain *i *interacting with a protein with domain *j *would result from false positives. Obtaining the multiple observations required for a high *E *score of erroneously inferred interacting domains will therefore be unlikely to occur by random experimental error.

Because DPEA detects only the domain interactions best supported by multiple observed protein interactions, we expect low sensitivity and high specificity in our predictions. DPEA's sensitivity may be impaired by the high rate of false negatives in existing interaction datasets, particularly in those organisms that have not been probed by high-throughput methods. Indeed, using the defined set of known positive and putative negative domain interactions in the PDB, we obtain a sensitivity of 6%. However, the specificity of 97% in the same test underscores the stringency of the *E *score. A more informative measure of DPEA's accuracy may be its positive predictive value of 70%, implying that roughly 2/3 of the high-confidence domain interactions inferred by DPEA are true positives; the remaining 1/3 are likely false positives. As interaction datasets become more complete, we expect the performance of DPEA to improve accordingly.

DPEA can be used to find domain interactions among families whose members interact highly specifically by screening for interactions with a low *θ* and a high *E*. This is in contrast to previously explored measures of domain-domain correlation, which were based on domains' inferred probability of interaction [23,24,27,28], and which are most likely to reward promiscuous, or low-specificity interactions (Figure 3a). Specificity is imperative for maintaining the fidelity of cellular signaling pathways in networks containing homologous interaction domains [45], and thus is of clear biological importance. DPEA is thus an extension of previous measures of domain-domain correlation in identifying highly specific domain interactions.

Our analysis of recurring domain interaction preferences in the multi-species data in the Database of Interacting Proteins suggests conserved patterns of domain interaction [6]. We have presented a method for extracting evidence of phylogenetically conserved domain interaction preferences from the incompletely mapped interactomes of multiple organisms, thus adding value to these datasets. Further high-throughput interaction studies and continued mining of the literature for protein interactions should continue to identify previously unrecognized domain interactions.

## Materials and methods

### Defining domains and their interactions

The July 2004 DIP full multispecies dataset was used. The DIP database represents protein interaction networks as a graph structure: proteins are nodes, and interactions between proteins are edges connecting the nodes (DIP proteins and their interactions are in Additional data files 2 and 3, respectively). For the 68 organisms we analyzed in DIP, a protein interaction network was defined consisting of all of each organism's proteins known to participate in an interaction with another protein also in that same organism, and the interactions between them. For simplicity, we did not include the 396 cross-species interactions in DIP.

For each organism, *τ*, that organism's observed network of interactions is defined as:



If we do not have experimental information demonstrating that two proteins interact, we assume that they do not interact. Therefore, for all taxa, *τ*, the interaction network is assumed to be incomplete: many biologically relevant interactions are surely unknown, and unreported in DIP. For simplicity in incorporating protein interaction data from multiple species, a pair of proteins is defined as potentially interacting if the proteins belong to the same organism. Thus,  is only defined when proteins *x *and *y *both belong to organism *τ*. All proteins *x *belong to one and only one organism, *τ*.

We then define the domains of each DIP protein (Additional data file 4). Pfam-A and -B domains were defined on DIP sequences in two ways. First, the DIP protein's SwissProt accession number, if available, was mapped to the domain annotations in the Pfam 14.0 version of the swisspfam file [46]. Second, DIP protein sequences were mapped to SwissProt [47] sequences using a BLAST search [48] with an *E*-value threshold of 10^-4^. Then, if an aligned segment of a SwissProt protein completely encompassed a Pfam domain as defined in the swisspfam file, the domain annotation was transferred to the DIP protein. Domain boundaries were allowed to overlap. By these two methods of domain mapping, 74% of amino acids in DIP proteins were assigned to an interval corresponding to a Pfam domain. The remaining 26% of amino acids remained unannotated, even though it is possible that some of these amino acids contain protein interaction sites.

In our model, we use the indices *i *and *j *to indicate domains and the indices *x *and *y *to indicate proteins. We define *D*(*x*) as an unordered collection of one or more domains on protein *x*. We do not consider multiple instances of a kind of domain on a protein, as in the case of WD40 domains; such a domain is only present once per protein in our model. Domains *i *and *j *are defined as potentially interacting if there exists at least one pair of interacting proteins *x *and *y *such that *i *∈ *D*(*x*) and *j *∈ *D*(*y*).

### Estimating probabilities of domain interactions by the EM algorithm

EM [29] is a numerical method for obtaining a maximum-likelihood estimate of some parameters of a model given incomplete data. The application of EM to inferring domain interactions from yeast two-hybrid protein interaction data has been explored previously by Deng *et al*. [24]. Here we extend the use of EM for estimating probabilities of each kind of potential domain interaction as a starting point for our analysis of the change in likelihood of a set of observed protein interactions, when a potential underlying domain interaction is excluded from the model.

We first obtain an estimate of *θ*_*ij*_, the multi-species probability of domains *i *and *j *interacting, that maximizes the likelihood of the observed protein interaction data. In our model, a given *θ*_*ij *_is the same for all species. This simplifies our computation, although it may not always be correct, as different organisms may use a given domain interaction to different extents.

We augment our observed data (protein-protein interactions and the domains on the proteins) with missing data (the unobserved domain-domain interactions) to obtain what is known in EM as the 'complete data'. To do this we iterate over all observed interacting protein pairs *x*,*y *in all organisms, and define all potential domain interactions underlying each observed protein interaction. The hidden domain interactions are represented in a data structure, ***C***:



***C ***is initialized by setting all  = 1. It is assumed that domain pairs interact independently and that multiple domain pairs may interact in the same protein pair. From ***C ***we define three statistics pertaining to the unobserved domain interactions:



is the number of interacting *i*,*j *domain pairs in interacting *x*,*y *proteins pairs.



is the number of non-interacting *i*,*j *domain pairs in interacting *x*,*y *proteins pairs.

*Z*_*ij *_is the number of non-interacting protein pairs with domain *i *in one protein and *j *in the other.

During EM, *M*_*ij *_and *N*_*ij *_will vary with the changing 0–1 values of . *Z*_*ij*_, however, remains constant because it is defined from unobserved protein interactions in ***O***.

For an initial estimate of *θ*_*ij*_, we calculate *S*_*ij*_, a measure of domain-domain correlation [23,27,28]:



From ***θ***and ***C ***we can now estimate a likelihood *L *of the observed protein interactions:



*α* and *β* are pseudocounts of arbitrary value, which in the present work were set to 1. The effect of the pseudocounts is to prevent *θ*_*ij *_from being exactly zero or one in the case of few instances of domains *i *and *j *in the data. Extremely high or low *θ*_*ij *_can therefore arise only from large numbers of observations pertaining to the potential interaction of domains *i *and *j*.

The EM algorithm proceeds as follows:

1. Find the expected value of all :



An important feature of this step is that, while  is a 0–1 variable, its expectation may have fractional values, dependent on ***θ***.

2. Use the expected value of  to compute the expected values of all *M*_*ij*_, and *N*_*ij*_:



3. Use the expected values of *M*_*ij*_, and *N*_*ij *_to re-estimate all *θ*_*ij*_:



4. Repeat until the likelihood, given by equation (1) no longer increases appreciably. 100 iterations of EM increased the log-likelihood function from -3.6 × 10^6 ^to -6.8 × 10^5^, showing the improved fit of our model to the data given the optimized values of ***θ***.

In summary, the observed protein interactions in DIP are held constant while the unobserved domain interactions are allowed to vary so as to maximize the likelihood of the observations, given in equation (1). This gives us ***θ***, a matrix of probabilities of domain interactions.

### Computing *E *scores

A measure of the evidence that domain *i *interacts with domain *j *is given by:



The numerator in the ratio in (2) is the probability that proteins *m *and *n *interact given that domains *i *and *j *might interact. The denominator is the probability that proteins *m *and *n *interact given that domains *i *and *j *do not interact. *E*_*ij *_is therefore a measure of the evidence that domains *i *and *j *ever interact.

To calculate *E*_*ij*_, we first compute the numerator in (3) using the maximum likelihood estimate of *θ* from EM. Then, to compute the denominator, we define , a new matrix of domain interaction probabilities with the same dimensions as *θ*, representing the same set of potential domain interactions. However, in , we set the probability of domains *i *and *j *interacting () to 0, then holding it at 0, rerun EM to allow competing domain interactions to maximize the likelihood of the observations in ***O***, under the model that domains *i *and *j *do not interact. This yields a maximum likelihood estimate of all possible domain interactions, in which all potential interactions of domains *i *and *j *are excluded (given a probability of 0), and which allows us to compute the denominator in (3).



The log of the resulting ratio is then summed across all organisms *τ* and all observed interacting protein pairs *x *and *y *potentially interacting through the domains *i *and *j*. If *i *and *j *are the only domains in proteins *x *and *y*, respectively, then the denominator is set to *ρ*, the background probability of any two proteins interacting, to prevent zero-division errors. *ρ* was set to 0.001 in this study.

An important feature of the *E *score is that more instances of domains *i *and *j *potentially interacting results in a higher *E*_*ij*_, consistent with the intuition that more observations of a kind of potential domain-domain interaction should increase the confidence in that interaction. Also, even for cases of low *θ*_*ij*_, a high *E*_*ij *_can result if *θ*_*ij *_is nonetheless high relative to competing .

The *E *score is calculated using only information on recorded interactions, hence it is not exactly equivalent to a standard likelihood ratio test, which would also consider unobserved interactions. The rationale behind this decision is that we do not wish to give excessive weight to negative interactions, as they are not documented in DIP. The *E *score instead aims to explain observed protein interactions in terms of the relative contributions of domain interactions to the likelihood of the observations.

### Validating inferred domain interactions

We confirmed inferred domain interactions using examples of interacting Pfam-A domains in the iPfam database [33]. Because we are validating domain interactions inferred from inter-chain protein interactions, only domain interactions that occur between chains in iPfam were used; domain interactions that only occur within chains were excluded.

To validate inferred domain interactions we first defined a DIP training set with the 230 protein interactions derived solely from X-ray diffraction experiments removed. We ran DPEA on this training set to analyze the evidence for 176,621 potentially interacting domain pairs. Mappings of Pfam-A domains to PDB structures, and the interactions of Pfam-A domains, were derived directly from the iPfam database tables. Potentially interacting domain pairs were ranked by three measures: *S*, *θ* and *E*. At various rank cutoffs the number of domain pairs known to interact in a protein complex in the PDB was counted. If a potentially interacting domain pair was found to interact in the PDB, it was considered a positive result. Because structural studies have sampled only a small fraction of biologically occurring domain interactions, the lack of a domain interaction in the PDB by itself cannot be taken to mean that a domain pair does not interact. Nevertheless, a reasonable domain-domain scoring strategy should include more structural interacting pairs in the highest ranked predictions than expected at random. Of the potentially interacting domain pairs in DIP, 0.4% also interacted in the PDB. Thus if, at a given rank cutoff, significantly more than 0.4% of the domain pairs interacted in the PDB, the method should be enriching for physically interacting domain pairs.

Significance was estimated using a binomial model:



*P *is the probability that, in a sample of domain pairs of size *n*, *i *or more pairs would be found in the PDB. *q *was set to 0.0040, the average frequency of PDB complexes in the potentially interacting domains in DIP.

To define a set of modular domain interactions, we filtered the set of domain interactions derived from DPEA of the DIP dataset with X-ray diffraction data to exclude any domain pair in which neither domain had *m *≥ 2. Thus, all domain pairs involved at least one modular domain. In total, 13% of the domains in DIP have *m *< 2.0 and the 2,157 interactions among any two of these domains were excluded. In all, the filtered set of inferred domain interactions included 174,464 domain pairs.

To define sets of known interacting and putative non-interacting domain pairs, we used the iPfam [33] tables to extract domain pairs that occur on separate chains in the same PDB complex. We excluded cases of two instances of the same domain interacting, and domain pairs that always occur as the only two domains in a PDB structure. We then separated the resulting domain pairs into two groups: those defined as interacting in the iPfam table int_pfamAs, and those not defined as interacting. This yielded a set of 295 known interacting and a set of 265 putative non-interacting domain pairs. Although the absence of an observed interaction between any pair of putative non-interacting domains does not mean that they never interact in nature, we assume that this set contains primarily domain pairs which do not interact.

Using a prediction threshold of *E *> 3.0 we defined interacting and putative non-interacting sets contain 18 true positives (TP), 7 false positives (FP), 258 true negatives (TN), and 277 false negatives (FN). Sensitivity and specificity of our predictions are calculated as follows: sensitivity = TP/(TP + FN); specificity = TN/(TN + FP). Positive predictive value is TP/(TP + FP) and can also be estimated from the relative enrichments of interacting and non-interacting domains in high-confidence predictions. We estimate sensitivity = 6%, specificity = 97%, and positive predictive value = 70%.

The same binomial significance test described above was used to assess enrichment of non-interacting domain pairs with high *E *scores.

### Defining domain modularity

Domains typically occur in proteins in combinations with other domains. Many modular domains are known to have a role in protein interactions [3]. It is therefore of interest to know which inferred interacting domains are modular, and which tend to occur as the only domain in a protein. To quantify the modularity of domain *i*, we calculated *m*_*i*_, the average number of domains occurring in proteins that contain domain *i*:



We mapped DIP proteins to both Pfam-A and -B domains, the latter of which are often short peptide motifs rather than proper domains in the classical sense. Therefore, some domains that occur as single-domain proteins, such as IL8 [Pfam:PF00048] [49,50] and Ras [Pfam:PF00071] [41] have a *m*_*i *_> 1.0, due to short Pfam-B domains occurring on the same protein as Pfam-A domains.

### Hypothetical protein network

We arbitrarily defined a hypothetical protein interaction network of appropriate format to analyze by DPEA (Additional data file 8). Possible domains were defined as a list of colors: red, violet, blue, azure, green, yellow, and orange. Proteins were initially defined as objects containing at least one domain. Proteins were then arbitrarily linked subject to the constraint that any interacting pair of proteins must contain a red domain in one protein and a blue domain in the other. We thus defined red ↔ blue as the underlying domain interaction in the network. This process was repeated for three organisms, arbitrarily chosen to be yeast, worm and human, with 5, 4 and 3 hypothetical proteins defined for each organism, respectively. DPEA was then applied to compute *S*, *θ* and *E *for all possible domain interactions in the network. Of these three scores, only *E *unambiguously identifies red ↔ blue as the underlying domain interaction in the hypothetical network (Figure 1).

### Augmenting yeast Cyclin_N ↔ Pkinase interactions

Our DIP dataset contains 11,593 interactions of yeast proteins. Of these, 26 are between proteins with Cyclin_N domains [Pfam:PF00134] and proteins with Pkinase domains [Pfam:PF00069]. To increase the number of interactions between these two classes of proteins by a factor of 3, we picked random pairs of proteins consisting of one member of each class and assigned an interaction if the interaction was not already in our data. This was repeated until the number of interactions between Cyclin_N-containing and Pkinase-containing proteins reached 78 (3 × 26). The DPEA algorithm was then run on both the unmodified DIP yeast interaction set, and the set with added interactions.

## Additional data files

The following additional data are included with the online version of this article: a table showing the numbers of DIP proteins and protein-protein interactions used per organism (Additional data file 1), a dataset of DIP proteins used in this study (Additional data file 2), a dataset of DIP interactions used in this study (Additional data file 3), a dataset of DIP-to-Pfam 14.0 mappings (Additional data file 4), a dataset of High-confidence inferred interacting domains in DIP (Additional data file 5), a dataset of DIP yeast interactions (Additional data file 6), a dataset of simulated false-negative interactions between yeast Cyclin_N- and Pkinase-containing proteins in DIP (Additional data file 7), and a dataset showing the hypothetical network from Figure 1 (Additional data file 8).

## Supplementary Material

Additional data file 1 Numbers of DIP proteins and protein-protein interactions used per organism Click here for file

Additional data file 2 DIP proteins used in this study Click here for file

Additional data file 3 DIP interactions used in this study Click here for file

Additional data file 4 DIP protein accession numbers followed by the Pfam 14.0 domain ID Click here for file

Additional data file 5 3,005 inferred domain interactions with an *E *score > 3.0, and the organisms that contributed evidence Click here for file

Additional data file 6 All protein interactions from the DIP dataset in Additional data file 3 in which both proteins are from the yeast *Saccharomyces. cerevisiae* Click here for file

Additional data file 7 52 randomly-assigned, unrecorded interactions between yeast proteins where one protein has a Cyclin_N domain and the other protein has a Pkinase domain. For all simulated interactions, the field exp_class is given the value 'h' for 'hypothetical' Click here for file

Additional data file 8 Nodes, edges, and node-to-domain mappings in a hypothetical protein interaction network. Format of each respective section is the same as in additional data files for DIP proteins, interactions, and domain mappings. Click here for file

## Figures and Tables

**Table 1:** High-confidence inferred domain interactions

Domain *I*				Domain *j*				Inferred interaction				
								
Pfam ID_*i*_	Pfam accession_*i*_	*n*_*i*_	*m*_*i*_	Pfam ID_*j*_	Pfam accession_*j*_	*n*_*j*_	*m*_*j*_	*S*_*ij*_	*θ*_*ij*_	*E*_*ij*_	Domains interact in PDB	Organisms providing evidence
LSM	PF01423	33	1.0	LSM	PF01423	33	1.0	0.18	0.174	387	x	Ce, Dm, Ec, Sc
IL8	PF00048	34	1.6	7tm_1	PF00001	44	1.7	0.12	0.070	139		Hs, Mm
Proteasome	PF00227	37	1.2	Proteasome	PF00227	37	1.2	0.076	0.060	103	x	Dm, Ec, Sc
Ferritin	PF00210	9	1.0	Ferritin	PF00210	9	1.0	0.35	0.360	47	x	Ce, Dm, Ec, Hp
Globin	PF00042	9	1.2	Globin	PF00042	9	1.2	0.37	0.381	42	x	Ai, Hs
EMP24_GP25L	PF01105	6	1.0	EMP24_GP25L	PF01105	6	1.0	0.33	0.350	35		Sc
CK_II_beta	PF01214	6	1.0	CK_II_beta	PF01214	6	1.0	0.63	0.600	32	x	Hs, Oc, Sc
Zf-C3HC4	PF00097	108	3.9	UQ_con	PF00179	39	1.1	0.017	0.011	29	x	Ce, Dm, Hs, Sc
WD40	PF00400	207	3.1	Cpn60_TCP1	PF00118	24	1.5	0.041	0.010	28		Dm, Sc
Cofilin_ADF	PF00241	9	1.9	Actin	PF00022	28	1.4	0.11	0.092	27		Dm, Sc
Ras	PF00071	69	1.8	Hrf1	PF03878	1	1.0	0.44	0.279	23		Sc
Lsm_interact	PF05391	1	2.0	LSM	PF01423	33	1.0	0.38	0.386	23		Sc
Pkinase	PF00069	399	3.7	Cyclin_N	PF00134	42	2.4	0.013	0.006	23	x	Ce, Dm, Hs, Mm, Sc, Sp
Bac_DNA_binding	PF00216	4	1.0	Bac_DNA_binding	PF00216	4	1.0	0.25	0.278	23	x	Ec
IF-2B	PF01008	7	1.0	IF-2B	PF01008	7	1.0	0.24	0.263	22		Sc
Clat_adaptor_s	PF01217	6	1.2	Adap_comp_sub	PF00928	8	2.2	0.20	0.227	22	x	Sc
Y_phosphatase2	PF03162	5	1.0	Y_phosphatase2	PF03162	5	1.0	0.16	0.185	21		Sc
LSM	PF01423	33	1.0	DIM1	PF02966	2	1.0	0.138	0.161	20		Sc
Zf-U1	PF06220	2	1.0	LSM	PF01423	33	1.0	0.138	0.161	20		Sc
Chorion_3	PF05387	2	1.0	CBM_14	PF01607	20	1.7	0.133	0.156	20		Dm
P5CR	PF01089	3	1.0	P5CR	PF01089	3	1.0	1.000	0.800	20		Dm, Hp, Sc
Tektin	PF03148	3	1.0	gamma-BBH	PF03322	3	1.0	1.000	0.800	20		Dm
P-II	PF00543	2	1.0	P-II	PF00543	2	1.0	0.750	0.667	20	x	Ec
HSP20	PF00011	18	1.2	HSP20	PF00011	18	1.2	0.041	0.048	19		Ce, Dm, Sc
Pfam-B_9658	PB009658	1	2.0	Histone	PF00125	19	1.8	0.571	0.555	19		Sc
TRAPP_Bet3	PF04051	4	1.0	Sybindin	PF04099	3	1.0	0.600	0.571	19		Ce, Sc
IF-2B	PF01008	7	1.0	DUF292	PF03398	2	1.0	0.600	0.571	19		Sc
Prenyltrans	PF00432	7	1.6	PPTA	PF01239	6	2.2	0.583	0.441	19	x	Dm, Rn, Sc
Glycogen_syn	PF05693	4	1.0	Glycogen_syn	PF05693	4	1.0	0.500	0.500	19		Sc
CBFD_NFYB_HMF	PF00808	13	1.4	CBFD_NFYB_HMF	PF00808	13	1.4	0.109	0.097	19	x	Dm, Rn, Sc
Ras	PF00071	69	1.8	GDI	PF00996	5	1.2	0.165	0.077	18		Mm, Sc
Cpn60_TCP1	PF00118	24	1.5	Cpn60_TCP1	PF00118	24	1.5	0.035	0.035	18	x	Dm, Ec, Sc, Ta
Porin_1	PF00267	3	1.0	Porin_1	PF00267	3	1.0	0.333	0.364	18	x	Ec
PNP_UDP_1	PF01048	3	1.0	PNP_UDP_1	PF01048	3	1.0	0.333	0.364	18	x	Ec
Prefoldin	PF02996	10	1.6	KE2	PF01920	10	1.3	0.323	0.237	18	x	Ce, Dm, Sc
Yip1	PF04893	4	1.0	Ras	PF00071	69	1.8	0.143	0.069	17		Sc
Autotransporter	PF03797	5	3.2	Autotransporter	PF03797	5	3.2	0.412	0.278	17		Ec, Hp
Chitin_bind_4	PF00379	35	1.3	Chitin_bind_4	PF00379	35	1.3	0.007	0.008	17		Dm
ATP_bind_1	PF03029	5	1.0	ATP_bind_1	PF03029	5	1.0	0.231	0.267	17		Ce, Sc
UQ_con	PF00179	39	1.1	Ubiquitin	PF00240	42	2.3	0.013	0.015	16		Hs, Sc
Pkinase	PF00069	399	3.7	CK_II_beta	PF01214	6	1.0	0.015	0.015	16	x	Dm, Hs, Sc
Ribosomal_S28e	PF01200	1	1.0	LSM	PF01423	33	1.0	0.188	0.222	16		Sc
Proteasome	PF00227	37	1.2	Pfam-B_57010	PB057010	2	3.0	0.464	0.434	16		Sc
RRM_1	PF00076	179	2.5	Pfam-B_4884	PB004884	3	1.3	0.049	0.038	16		Dm, Sc
Profilin	PF00235	3	1.0	Actin	PF00022	28	1.4	0.150	0.182	16		Bt, Dm, Sc
Adap_comp_sub	PF00928	8	2.2	Adaptin_N	PF01602	17	2.6	0.182	0.122	15	x	Sc
vATP-synt_AC39	PF01992	2	1.0	adh_short	PF00106	30	1.3	0.125	0.154	15		Sc
Rho_GDI	PF02115	1	1.0	Ras	PF00071	69	1.8	0.120	0.148	15	x	Sc
Pfam-B_4092	PB004092	1	2.0	LIM	PF00412	37	2.4	0.238	0.257	15		Dm
ADH_zinc_N	PF00107	29	1.6	ADH_zinc_N	PF00107	29	1.6	0.016	0.019	15	x	Ec, Sc

Domain pairs are ranked by their *E *score. For domain *i*, *n*_*i *_is the number of DIP proteins that contain domain *i*; *m*_*i *_is the average number of domains in a protein that contains domain *i*. Domain pairs known to interact in PDB complexes are marked with an 'x'. Organisms whose protein interaction data provided evidence for each domain interaction are given. Ai, *Anser indicus *(Bar-headed goose); Bt, *Bos taurus*; Ce, *Caenorhabditis elegans*; Dm, *Drosophila melanogaster*, Ec, *Escherichia coli*; Hp, *Helicobacter pylori *26695; Hs, *Homo sapiens*; Mm, *Mus musculus*; Oc, *Oryctolagus cuniculus*; Rn, *Rattus norvegicus*; Sc, *Saccharomyces cerevisiae*; Sp, *Schizosaccharomyces pombe*; Ta, *Thermoplasma acidophilum*.

**Table 2:** Potential domain interactions of G-protein β and γ subunits

G-protein γ subunit domain		G-protein β subunit domain			
			
Pfam ID	Pfam accession	Pfam ID	Pfam accession	*θ*	*E*
G-gamma	PF00631	Pfam-B_2804	PB002804	0.83	12
G-gamma	PF00631	Pfam-B_17462	PB017462	0.62	0.44
G-gamma	PF00631	Pfam-B_92195	PB092195	0.56	0.17
G-gamma	PF00631	Pfam-B_12983	PB012983	0.56	0.17
G-gamma	PF00631	WD40	PF00400	0.008	0.003

In a network of interacting G-γ and G-β proteins, the interaction of domains G-gamma [Pfam:PF00631] and a Pfam-B domain found at the N-terminus of some G-β proteins [Pfam:PB002804] is the only domain interaction out of several possibilities with an *E *score > 3.0. This inferred domain interaction is consistent with structural [37] and biochemical [38,39] evidence that the G-β N-terminal region corresponding to PB002804 is important for the formation of G-βγ complexes, and with the observation that many other proteins with WD40 domains do not interact with proteins of the G-gamma domain family (data not shown). Apparently DPEA identifies domain interactions in G-βγ complexes.

**Table 3:** Potential domain interactions between Ran homologs and Ran-binding proteins

Ran homolog domain		Ran-binding protein domain			
			
Pfam ID	Pfam accession	Pfam ID	Pfam accession	*θ*	*E*
Pfam-B_1470	PB001470	Ran_BP1	PF00638	0.31	3.6
Pfam-B_1470	PB001470	Pfam-B_102314	PB102314	0.42	0.46
Pfam-B_1470	PB001470	Pfam-B_5293	PB005293	0.14	0.19
Pfam-B_1470	PB001470	Pfam-B_93946	PB093946	0.55	0.19
Pfam-B_1470	PB001470	Pfam-B_87101	PB087101	0.20	0.19
Pfam-B_1470	PB001470	Pfam-B_73892	PB073892	0.35	0.075
Ras	PF00071	Pfam-B_102314	PB102314	0.039	0.018
Ras	PF00071	Pfam-B_93946	PB093946	0.092	0.014
Ras	PF00071	Ran_BP1	PF00638	0.008	0.011
Ras	PF00071	Pfam-B_87101	PB087101	0.013	0.009
Ras	PF00071	Pfam-B_5293	PB005293	0.008	0.008
Pfam-B_1470	PB001470	zf-RanBP	PF00641	0.053	0.008
Ras	PF00071	Pfam-B_73892	PB073892	0.029	0.004
Ras	PF00071	zf-RanBP	PF00641	0.004	0.000

Several domain interactions are possible in the interactions of yeast and worm Ran signal-transducing proteins with some Ran-binding proteins. Of these possible domain interactions, DPEA predicts the interaction (*E *= 3.6) of a Pfam-B domain [Pfam:PB001470] found at the C-termini of Ran homologs but not in other Ras family members [Pfam:PF00071] with Ran-binding domains [Pfam:PF00638, Ran_BP1]. Structural [43] and biochemical [44] studies confirm this interaction. We conclude that DPEA identified, from among several possibilities, an important domain interaction for the interaction of Ran homologs with a subset of Ran-binding proteins.

## References

[B1] Yu H, Greenbaum D, Xin Lu H, Zhu X, Gerstein M (2004). Genomic analysis of essentiality within protein networks.. Trends Genet.

[B2] Eisenberg D, Marcotte EM, Xenarios I, Yeates TO (2000). Protein function in the post-genomic era.. Nature.

[B3] Pawson T, Nash P (2003). Assembly of cell regulatory systems through protein interaction domains.. Science.

[B4] McGough AM, Staiger CJ, Min JK, Simonetti KD (2003). The gelsolin family of actin regulatory proteins: modular structures, versatile functions.. FEBS Lett.

[B5] Lim WA, Richards FM, Fox RO (1994). Structural determinants of peptide-binding orientation and of sequence specificity in SH3 domains.. Nature.

[B6] Pereira-Leal JB, Teichmann SA (2005). Novel specificities emerge by stepwise duplication of functional modules.. Genome Res.

[B7] Sudol M (1998). From Src homology domains to other signaling modules: proposal of the 'protein recognition code'.. Oncogene.

[B8] Uetz P, Giot L, Cagney G, Mansfield TA, Judson RS, Knight JR, Lockshon D, Narayan V, Srinivasan M, Pochart P (2000). A comprehensive analysis of protein-protein interactions in *Saccharomyces cerevisiae*.. Nature.

[B9] Gavin AC, Bosche M, Krause R, Grandi P, Marzioch M, Bauer A, Schultz J, Rick JM, Michon AM, Cruciat CM (2002). Functional organization of the yeast proteome by systematic analysis of protein complexes.. Nature.

[B10] Giot L, Bader JS, Brouwer C, Chaudhuri A, Kuang B, Li Y, Hao YL, Ooi CE, Godwin B, Vitols E (2003). A protein interaction map of *Drosophila melanogaster*.. Science.

[B11] Li S, Armstrong CM, Bertin N, Ge H, Milstein S, Boxem M, Vidalain PO, Han JD, Chesneau A, Hao T (2004). A map of the interactome network of the metazoan *C. elegans*.. Science.

[B12] Butland G, Peregrin-Alvarez JM, Li J, Yang W, Yang X, Canadien V, Starostine A, Richards D, Beattie B, Krogan N (2005). Interaction network containing conserved and essential protein complexes in *Escherichia coli*.. Nature.

[B13] Chothia C, Gough J, Vogel C, Teichmann SA (2003). Evolution of the protein repertoire.. Science.

[B14] Tong AH, Drees B, Nardelli G, Bader GD, Brannetti B, Castagnoli L, Evangelista M, Ferracuti S, Nelson B, Paoluzi S (2002). A combined experimental and computational strategy to define protein interaction networks for peptide recognition modules.. Science.

[B15] Wang Z, Moult J (2001). SNPs, protein structure, and disease.. Hum Mutat.

[B16] Resch A, Xing Y, Modrek B, Gorlick M, Riley R, Lee C (2004). Assessing the impact of alternative splicing on domain interactions in the human proteome.. J Proteome Res.

[B17] Loregian A, Marsden HS, Palu G (2002). Protein-protein interactions as targets for antiviral chemotherapy.. Rev Med Virol.

[B18] Zutshi R, Brickner M, Chmielewski J (1998). Inhibiting the assembly of protein-protein interfaces.. Curr Opin Chem Biol.

[B19] Dueber JE, Yeh BJ, Bhattacharyya RP, Lim WA (2004). Rewiring cell signaling: the logic and plasticity of eukaryotic protein circuitry.. Curr Opin Struct Biol.

[B20] Salwinski L, Miller CS, Smith AJ, Pettit FK, Bowie JU, Eisenberg D (2004). The Database of Interacting Proteins: 2004 update.. Nucleic Acids Res.

[B21] Peri S, Navarro JD, Amanchy R, Kristiansen TZ, Jonnalagadda CK, Surendranath V, Niranjan V, Muthusamy B, Gandhi TK, Gronborg M (2003). Development of human protein reference database as an initial platform for approaching systems biology in humans.. Genome Res.

[B22] Wojcik J, Schachter V (2001). Protein-protein interaction map inference using interacting domain profile pairs.. Bioinformatics.

[B23] Sprinzak E, Margalit H (2001). Correlated sequence-signatures as markers of protein-protein interaction.. J Mol Biol.

[B24] Deng M, Mehta S, Sun F, Chen T (2002). Inferring domain-domain interactions from protein-protein interactions.. Genome Res.

[B25] Nye TM, Berzuini C, Gilks WR, Babu MM, Teichmann SA (2005). Statistical analysis of domains in interacting protein pairs.. Bioinformatics.

[B26] Database of Interacting Proteins. http://dip.doe-mbi.ucla.edu.

[B27] Kim WK, Park J, Suh JK (2002). Large scale statistical prediction of protein-protein interaction by potentially interacting domain (PID) pair.. Genome Inform Ser Workshop Genome Inform.

[B28] Ng SK, Zhang Z, Tan SH (2003). Integrative approach for computationally inferring protein domain interactions.. Bioinformatics.

[B29] Dempster AP, Laird NM, Rubin DB (1977). Maximum likelihood from incomplete data via EM algorithm.. J Royal Stat Soc, Series B.

[B30] The Protein Data Bank. http://www.rcsb.org/pdb/.

[B31] Berman HM, Westbrook J, Feng Z, Gilliland G, Bhat TN, Weissig H, Shindyalov IN, Bourne PE (2000). The Protein Data Bank.. Nucleic Acids Res.

[B32] Bateman A, Coin L, Durbin R, Finn RD, Hollich V, Griffiths-Jones S, Khanna A, Marshall M, Moxon S, Sonnhammer EL (2004). The Pfam protein families database.. Nucleic Acids Res.

[B33] Finn RD, Marshall M, Bateman A (2005). iPfam: visualization of protein-protein interactions in PDB at domain and amino acid resolutions.. Bioinformatics.

[B34] Zheng N, Wang P, Jeffrey PD, Pavletich NP (2000). Structure of a c-Cbl-UbcH7 complex: RING domain function in ubiquitin-protein ligases.. Cell.

[B35] Brown NR, Noble ME, Endicott JA, Johnson LN (1999). The structural basis for specificity of substrate and recruitment peptides for cyclin-dependent kinases.. Nat Cell Biol.

[B36] von Mering C, Krause R, Snel B, Cornell M, Oliver SG, Fields S, Bork P (2002). Comparative assessment of large-scale data sets of protein-protein interactions.. Nature.

[B37] Wall MA, Coleman DE, Lee E, Iniguez-Lluhi JA, Posner BA, Gilman AG, Sprang SR (1995). The structure of the G protein heterotrimer Gi alpha 1 beta 1 gamma 2.. Cell.

[B38] Garritsen A, van Galen PJ, Simonds WF (1993). The N-terminal coiled-coil domain of beta is essential for gamma association: a model for G-protein beta gamma subunit interaction.. Proc Natl Acad Sci USA.

[B39] Pellegrino S, Zhang S, Garritsen A, Simonds WF (1997). The coiled-coil region of the G protein beta subunit. Mutational analysis of Ggamma and effector interactions.. J Biol Chem.

[B40] Yan K, Kalyanaraman V, Gautam N (1996). Differential ability to form the G protein betagamma complex among members of the beta and gamma subunit families.. J Biol Chem.

[B41] Colicelli J (2004). Human RAS superfamily proteins and related GTPases.. Sci STKE.

[B42] Macara IG (2002). Why FRET about Ran?. Dev Cell.

[B43] Vetter IR, Nowak C, Nishimoto T, Kuhlmann J, Wittinghofer A (1999). Structure of a Ran-binding domain complexed with Ran bound to a GTP analogue: implications for nuclear transport.. Nature.

[B44] Kuhlmann J, Macara I, Wittinghofer A (1997). Dynamic and equilibrium studies on the interaction of Ran with its effector, RanBP1.. Biochemistry.

[B45] Zarrinpar A, Park SH, Lim WA (2003). Optimization of specificity in a cellular protein interaction network by negative selection.. Nature.

[B46] Swisspfam.gz. ftp://ftp.genetics.wustl.edu/pub/Pfam/swisspfam.gz.

[B47] Gasteiger E, Gattiker A, Hoogland C, Ivanyi I, Appel RD, Bairoch A (2003). ExPASy: The proteomics server for in-depth protein knowledge and analysis.. Nucleic Acids Res.

[B48] Altschul SF, Gish W, Miller W, Myers EW, Lipman DJ (1990). Basic local alignment search tool.. J Mol Biol.

[B49] Clore GM, Gronenborn AM (1995). Three-dimensional structures of alpha and beta chemokines.. FASEB J.

[B50] Loetscher P, Clark-Lewis I (2001). Agonistic and antagonistic activities of chemokines.. J Leukoc Biol.

