# Supplementary material for: Inferring protein domain interactions from databases of interacting proteins
Source: Genome Biol. 2005 Sep 19;6(10):R89. doi: 10.1186/gb-2005-6-10-r89 (PMC1257472; doi:10.1186/gb-2005-6-10-r89)
Supplement: Additional data file 1 — Numbers of DIP proteins and protein-protein interactions used per organism [file gb-2005-6-10-r89-S1.pdf]

Organism	Proteins	Protein-protein interactions
Saccharomyces cerevisiae	3476	11593
Drosophila melanogaster	3777	8738
Caenorhabditis elegans	2233	3362
Homo sapiens	799	1037
Escherichia coli	417	505
Helicobacter pylori 26695	318	488
Mus musculus	129	112
Rattus norvegicus	54	40
Vaccinia virus	37	31
Bos taurus	11	11
Bacillus subtilis	9	9
Pisum sativum	7	8
Schizosaccharomyces pombe	10	7
human herpesvirus 1	7	6
Klebsiella aerogenes	3	5
Oryctolagus cuniculus	4	5
Mycobacterium tuberculosis	5	4
Salmonella typhimurium	3	3
plasmid RK2	2	3
Thermoplasma acidophilum	2	3
Neurospora crassa	2	3
Paracoccus denitrificans	6	3
Spinacia oleracea (Spinach)	2	3
Anser indicus (Bar-headed goose)	2	3
Thermus aquaticus	4	2
Hordeum vulgare	3	2
Gallus gallus	4	2
Thermotoga maritima	2	2
Lathyrus ochrus	3	2
bovine respiratory syncytial virus	3	2
Klebsiella pneumoniae	2	1
Pseudomonas putida	2	1
Propionibacterium freudenreichii subsp. shermanii	2	1
Desulfovibrio fructosovorans	2	1
Sulfolobus solfataricus	2	1
Enterobacteria phage G4	2	1
Allochromatium vinosum	2	1
Alcaligenes eutrophus	2	1
Rous sarcoma virus	1	1
Coliphage phiX174	2	1
Rhodococcus sp.	2	1
Azotobacter vinelandii	1	1
Gadus callarias	1	1

Acidaminococcus fermentans	2	1
Escherichia coli (R483 plasmid)	1	1
Human adenovirus type 5	2	1
Bombyx mori	2	1
Xenopus laevis	2	1
Drosophila hydei	1	1
Enterobacteria phage T7	2	1
Thermus sp.	2	1
Corynebacterium glutamicum	1	1
bunyamwera virus	1	1
Agrobacterium tumefaciens	2	1
Geobacillus stearothermophilus	2	1
Shigella dysenteriae	2	1
parainfluenza virus type 3	2	1
Polyomavirus muris	2	1
Haemophilus influenzae	2	1
Oxytricha nova	2	1
Desulfovibrio vulgaris	2	1
Desulfovibrio baculatus	2	1
thermophilic bacterium PS3	2	1
Bacillus amyloliquefaciens	2	1
Synechococcus sp.	2	1
Pseudomonas sp.	2	1
Clostridium botulinum	2	1
Vibrio cholerae	2	1
Total	11403	26032
